# Total phenolic distribution of juice, peel, and seed extracts of four pomegranate cultivars

**DOI:** 10.4103/0973-1296.80681

**Published:** 2011

**Authors:** Şadiye Gözlekçi, Onur Saraçoğlu, Ebru Onursal, Mustafa Özgen

**Affiliations:** *Department of Horticulture, Akdeniz University Agricultural Faculty, 07058 Antalya, Turkey*; 1 *Department of Horticulture, Gaziosmanpasa University Agricultural Faculty, 60240 Tokat, Turkey*

**Keywords:** Bioactive compounds, fruit, phenolics, *Punica granatum L*

## Abstract

The total phenolic distribution of juice, peel, and seed extracts of four Turkish pomegranate, *Punica granatum* L., cultivars (“Lefan,” “Katirbasi,” “Cekirdeksiz-IV,” and “Asinar”) was investigated. Total phenolic compounds were determined with the Folin–Ciocalteu colorimetric method. The results showed that the levels of total phenolic compounds changed depending on cultivars and fruit parts. In all cultivars, the highest levels of total phenolic content were obtained from the peel extracts. The total phenolic content ranged from 1775.4 to 3547.8 mg gallic acid equivalent (GAE)/L among the cultivars. However, the total phenolic content of pomegranate juice and seed extract ranged from 784.4 to 1551.5 mg GAE/L and 117.0 to 177.4 mg GAE/L, respectively. “Lefan” displayed the highest amount of the total phenolic content among the four popular cultivars tested.

## INTRODUCTION

Pomegranate (*Punica granatum* L.) is an ancient fruit with exceptionally rich ethnomedical applications. The peel (pericarp) is well regarded for its astringent properties; the seeds for conferring invulnerability in combat and stimulating beauty and fertility.[1] Pomegranates have been used extensively in the folk medicine of many cultures.[2] It is an ancient fruit with an illustrious medical history and has been the subject of classical reviews for over 100 years.[3] Several researchers reported that modern uses of pomegranate-derived products now include prevention and treatment of some of the cancer types such as lung cancer[4] and prostate cancer.[5]

According to Lansky and Newman,[6] over the past few decades scientific investigations have laid a credible basis for some of the traditional ethnomedical uses of the pomegranate. These studies, most completed in the past 5 years, may be divided into several general areas. For instance, the pomegranate-mediated antioxidant activity can be considered a means of lowering the threshold for inflammation. The antioxidant activity as well as suppression of inflammation may contribute to chemotherapeutic and chemopreventive utility against cancer. The potent antioxidant activities of pomegranates are attributed to its polyphenols.[7] Phenolic compounds are important components of many fruits.[8] As Robards *et al*.[9] reported, there are approximately 5000 known plant phenolics and model studies have demonstrated that many of them have antioxidant activities. The antioxidant activity of phenolics is mainly due to their redox properties, which allow them to act as reducing agents, hydrogen donors, singlet oxygen quenchers, and metal chelators.[10]

Pomegranate is one of the oldest cultivated species with a high genetic diversity.[11] This fruit is cultivated extensively in Iran, Afghanistan, India, Mediterranean countries, and Turkey. Due to the long historic cultivation and suitable climatic conditions of Turkey, there are numerous different pomegranate cultivars available with different peel and aril color, aroma, taste etc., for fresh and processing market. The objective of this study was to determine and compare the total phenolic compounds of peel, aril juice, and seeds of four popular pomegranate cultivars grown in Turkey.

## MATERIALS AND METHODS

### Plant material and extraction

In this study, “Lefan,” “Katirbasi,” “Cekirdeksiz-IV,” and “Asinar” pomegranate cultivars, some of the most popular cultivars grown in Turkey, were harvested at commercial maturity at the Research Station of the Faculty of Agriculture, Akdeniz University, Antalya, Turkey. Arils of fruits were hand separated (about 100 g lots) and frozen at –20°C. Three replicates were maintained for each analysis, each replicate indicating four pomegranate fruits. Pomegranate juice was obtained from pomegranate arils by a hand press. The peels and seeds were manually removed, sun-dried, and homogenized for phenolic extractions.

The rind color of pomegranates was determined using a Minolta portable chromameter (model CR-400; Minolta, Kyoto, Japan) which provided CIE L^*^, a^*^, and b^*^ values. The chromameter describes color in three coordinates: L^*^, lightness, from 0 (black) to 100 (white); a^*^, from –60 (green) to 60 (red); and b^*^, from –60 (blue) to 60 (yellow). Chroma values was calculated as chroma (*a^2^+ b^2^*)^1/2^.

Also, three subsamples of 10 fruits were used for measurements of horticultural attributes such as fruit weight (g), fruit length (mm), fruit diameter (mm), shape index, aril weight (g), seed weight, fruit juice yield (%), and rind thickness (mm). Total soluble solid (TSS) content was determined with a digital refractometer (Atago, model ATC-1E, Kyoto, Japan). The total acidity (TA) was measured by titration with 0.1 N NaOH.

### Determination of total phenolics

Five gram of each sample was homogenized in 25 mL of the 50% (v/v) ethanol/water solution. The extract was filtered through the Whatman no. 41 filter paper for the removal of peel and seed particles. Only the peel extracts were diluted 10 times using distilled water. After filtration, the concentrations of total phenolics (TP) in extracts were determined by the Folin-Ciocalteu colorimetric method.[12] Samples (100 µL) were mixed with 5 mL of the 0.2 N Folin-Ciocalteu reagent and 4 mL of 7.5% sodium carbonate. The mixture was allowed to stand for 2 h at room temperature in the dark before the absorbance was measured at 765 nm spectrophotometrically. Gallic acid standards at eight different concentrations ranging from 100 to 2000 mg/L were prepared. By using these standards, the GA calibration curve was obtained and the total phenolic content was calculated from this curve and expressed as mg gallic acid equivalents (mg GAE/L).

### Statistical analysis

The estimation of the total phenolic content in the extracts was carried out in triplicates and differences among the means were determined for significance at *P*<0.05 using Duncan’s multiple range test and the system program SAS software package.[13]

## RESULTS AND DISCUSSION

The results indicated that there were significant differences among the cultivars for the variables tested. Table 1 presents the mean values obtained for fruit quality characteristic of the analyzed pomegranate cultivars. The aril color ranged from pink to dark red. “Katirbasi” with a 378 g fruit size had the largest fruits among the others. Other than shape index, all the other horticultural characteristics were statistically significant between the four cultivars.

**Table 1:** Some of the horticultural characteristics of pomegranate cultivars

Characteristics	Lefan	Katirbasi	Cekirdeksiz -IV	Asinar
Aril color	Light red	Red	Pink	Dark red
Taste	Soursweet	Soursweet	Sweet	Soursweet
Fruit weight (g)	377.89^b^	504.15^a^	398.79^b^	488.28^a^
Fruit length (mm)	78.94^b^	89.17^a^	80.95^b^	86.13^a^
Fruit diameter (mm)	91.66bc	100.06a	90.15c	95.70ab
Shape index (Fd/Fl)ns	1.17	1.12	1.11	1.12
Total aril weight/ fruit (g)	171.02b	253.55^a^	178.80^b^	258.08^a^
100 – Aril weight (g)	35.56^c^	45.29^a^	39.04^b^	44.58^a^
Seed weight/ fruit (g)	38.58^b^	47.17^a^	36.88^b^	49.31^a^
Fruit juice/fruit (g)	134.84^b^	211.86^a^	141.93^b^	208.77^a^
Fruit juice (%)	37.16^b^	48.32^a^	47.43^a^	48.69^a^
Rind thickness (mm)	3.62^a^	2.87^bc^	2.57^c^	3.29^ab^

Means were compared by Duncan’s multiple range test *(P<0.05)* within parametersin each line; means with the same letter do not differ significantly (^ns^ : not significant)

The soluble solid content of analyzed cultivars varied from 13.90% to 14.97%. “Cekirdeksiz-IV” and “Katirbasi” displayed the highest TSS (14.97% and 14.82%, respectively). In other studies, the TSS content varied from 14.4% to 16.2%,[14] 14.3% to 16.4%,[15] 14.1% to 16.5%,[16] and 14.7% to 17.9%.[17] On the other hand, titratable acidity of our cultivars ranged from 0.97% to 1.39%. However, there was no statistical difference among the cultivars regarding titratable acidity.

The peel colors were measured using a colorimeter and the “L^*^,” “a^*^,” “b^*^,” and *“C”* values were recorded [Table 2]. The highest value of the peel lightness (*L* value) was obtained from “Cekirdeksiz-IV” (94.26) and “Lefan” (91.76) cultivars. The fruit color of all the cultivars ranged from greenish yellow to pinkish red. Especially the color of “Lefan” cultivar was darker red than all the other cultivars. That’s why redness indication of a* values was the highest in “Lefan” (40.06), followed by “Katirbasi” (30.39). “Lefan” had the lowest (22.37) b* values indicating the color change from yellow to blue. “Asinar” had the lowest chroma values among the other cultivars.

**Table 2:** Pomological characteristics of pomegranate cultivars

Characteristics	Lefan	Katirbasi	Cekirdeksiz-IV	Asinar
TSS (%)	14.08^ab^	14.82^a^	14.97^a^	13.90^b^
TA (%)^ns^	0.98	0.97	0.99	1.39
L^*^	91.76^a^	60.12^b^	94.26^a^	46.46^c^
a^*^	40.06^a^	30.39^ab^	11.45^c^	19.64^bc^
b^*^	22.37^b^	29.79^a^	35.69^a^	33.17^a^
Chroma	33.67^a^	30.29^a^	36.20^a^	22.87^b^

Means were compared by Duncan’s multiple range test (*P*<0.05) within parameters in each line; means with the same letter do not differ significantly

(^ns^ : not significant)

The total phenolic contents of the peel, juice, and seed from pomegranate cultivars are presented in Table 3. The results indicated that pomegranate peel displayed the highest amount of fruit total phenolic content, (2747 mg/L) 67% of the total amount. Fruit juice and seed contained 29.7% and 3.3% of the total fruit phenolic content, respectively. These results are in agreement with the literature; Li *et al*.[18] found that pomegranate peel had the highest antioxidant activity among the peel, Pulp, and seed fractions of 28 kinds of fruits. They also identified that the total phenolic content of the peel extract was nearly 10-fold as high as that of the Pulp extract in pomegranate. A similar result was reported by Tomas-Barberan *et al*.[19] who found that peel tissues usually contained larger amount of phenolics than did flesh tissues. Also, Singh *et al*.[20] identified that pomegranate peel extracts exhibited a higher antioxidant activity in various *in vitro* models compared to seed extracts. It is known that these activities related to phenolic compounds. Most of the phenolic materials that are present in the pomegranate peel are passed onto the juice during pressing.[21]

**Table 3:** Total phenolic distribution of peel, juice, and seed in four pomegranate cultivars

Cultivar	Peel	Juice	Seed	Total
Lefan	3547.8^a^	1551.5^a^	125.3^b^	5224.6^a^
Katirbasi	3127.0^b^	1229.5^c^	121.2^c^	4477.8^b^
Cekirdeksiz-IV	2537.1^c^	784.4^d^	117.0^d^	3438.5^c^
Asinar	1775.4^d^	1307.3^b^	177.4^a^	3260.1^d^
Mean	2746.8	1218.2	135.2	4100.3

Total phenolic contents were estimated by the Folin–Ciocalteu assay of Singleton and Rossi.[12] Values are expressed as µg GAE/g fw. Means were compared by Duncan’s multiple range test (*P*<0.05) within parameters in each column; means with the same letter do not differ significantly (^ns^ : not significant)

The highest total phenolic content 3547 mg/L was found in the peel of “Lefan” followed by the peels of “Katirbasi” (3127 mg/L), “Cekirdeksiz-IV” (2537 mg/L), and “Asinar” (1775 mg/L) cultivars. The highest total phenolic content value for fruit juices (1551.5 mg/L) was found in “Lefan” followed by “Asinar” (1307.3 mg/L) and “Katirbasi” (1229.5 mg/L). There were significant statistical differences between the cultivars. Ozgen *et al*.[17] reported that the total phenolic content of six cultivars varied between 1245 and 2076 mg GAE/L of fruit juice. Karadeniz *et al*.[22] also found the total phenolic content in pomegranate juice to be 2408 mg/kg. Similar results for the juice were reported by Maiman and Ahmad.[23] It is observed that pomegranate seed extracts had the lowest total phenolic content in comparison with other fruit parts. The total phenolic content of the seeds from cultivars “Asinar,” “Lefan,” “Katirbasi,” and “Cekirdeksiz-IV” was 177.4 mg/L, 125.3 mg/L, 121.2 mg/L, and 117.0 mg/L, respectively. A similar result was obtained by Rosenblat and Aviram (2006), as a whole fruit total phenolic content. “Lefan” showed the highest total phenolic content (5224.6 mg/L), followed by “Katirbasi” (4477.8 mg/L), “Cekirdeksiz-IV”(3438.5 mg/L), and “Asinar” (3260.1 mg/L) cultivars.

## CONCLUSION

The results indicated that the total phenolic contents of pomegranate vary considerably from one cultivar to another. In addition, the concentration of phenolics varied depending on different parts of the fruit. Aril color and peel of fruits were responsible for the majority of the total phenolic content of pomegranate. Cultivars with a dark red color peel and aril displayed a higher phenolic content.
